# The Impact of an Elevated Uric Acid Level on the Prevalence of Coronary Artery Disease in Pancreas Transplant Candidates with Type 1 Diabetes: A Cross Sectional Study

**DOI:** 10.3390/jcm11092421

**Published:** 2022-04-26

**Authors:** Małgorzata Buksińska-Lisik, Przemysław Kwasiborski, Robert Ryczek, Wojciech Lisik, Artur Mamcarz

**Affiliations:** 1 3rd Department of Internal Medicine and Cardiology, Medical University of Warsaw, 04-749 Warsaw, Poland; artur.mamcarz@wum.edu.pl; 2 Department of Cardiology and Internal Diseases, Regional Hospital in Miedzylesie, 04-749 Warsaw, Poland; pkwasiborski77@gmail.com; 3 Department of Cardiology and Internal Diseases, Military Institute of Medicine, 04-141 Warsaw, Poland; rryczek@wim.mil.pl; 4 Department of General and Transplantation Surgery, Medical University of Warsaw, 02-006 Warsaw, Poland; wojciech.lisik@wum.edu.pl

**Keywords:** coronary artery disease, cardiovascular risk, hypertension, type 1 diabetes, pancreas transplantation, simultaneous pancreas-kidney transplantation, uric acid, hyperuricemia

## Abstract

Pancreas transplantation is considered a high-risk surgery with cardiovascular complications. Early detection of all potential cardiovascular risk factors can decrease the perioperative risk and improve the pancreas recipients’ outcome. The present study aims to evaluate the association between serum uric acid (UA) levels and the prevalence of coronary artery disease (CAD) in patients eligible for pancreas transplantation. We prospectively enrolled 63 consecutive patients with type 1 diabetes (T1D) who underwent cardiological evaluation before pancreas transplantation in our center. Participants underwent clinical evaluation, laboratory assays, and coronary angiography. The median concentration of UA in patients with CAD was significantly higher than in participants without CAD (6.43 (4.93–7.26) vs. 4.41 (3.64–5.49) mg/dL, *p* = 0.0002). We showed the positive correlation between UA concentration and systolic blood pressure, pulse pressure (PP) and triglycerides (r = 0.271, *p* = 0.032; r = 0.327, *p* = 0.009; r = 0.354, *p* = 0.004, respectively). In a multivariate analysis, the concentration of UA (OR 2.044; 95% CI: 1.261–3.311, *p* = 0.004) was independently associated with the prevalence of CAD in pancreas transplant candidates with T1D. We demonstrated that elevated UA levels were strongly associated with the high prevalence of CAD in pancreas transplant candidates with T1D. To stratify cardiovascular risk, the measurement of the UA concentration should be considered in all T1D patients qualified for pancreas transplantation.

## 1. Introduction

Pancreas transplantation is a widely accepted method of treatment for selected patients with type 1 diabetes (T1D) that provides sustained glycemic control and improves the prognosis [1,2,3]. However, cardiovascular complications occurring in about 16.3–30% of pancreas recipients are one of the more common causes of early and late death of pancreas transplant recipients [4,5]. This is mainly due to a high prevalence of coronary artery disease (CAD) that is diagnosed in about 19–47.5% of pancreas transplant candidates, despite being asymptomatic [6,7,8]. The exacerbate progression of atherosclerosis is the effect of chronic hyperglycemia [9]. Moreover, the role of common cardiovascular disease risk factors (CVD) (hypertension, hyperlipidemia, smoking) is also important [10,11]. In addition to the traditional risk factors, the role of additional CVD risk factors is gaining attention. Hyperuricemia is one of such non-conventional risk factors for CVD [12,13].

Hyperuricemia is typically defined as increased serum uric acid concentrations above 7 mg/dL in men and above 6 mg/dL in women [14]. The prevalence of hyperuricemia affects approximately 20–25% of the general population [15,16]. The relationship between hyperuricemia and coronary heart disease has been widely discussed since Gertler et al. introduced uric acid (UA) as a potential risk factor for coronary heart disease [17]. Numerous studies have linked hyperuricemia to cardiovascular disease; elevated UA correlates with a higher risk of stroke [18,19], carotid atherosclerosis [20], hypertension [21,22], CAD [23,24], and CAD-related mortality [25].

The diagnostic thresholds for high UA levels in terms of cardiovascular risk are considered to be lower than those associated with gout [26]. Higher levels of UA lead to an increased CVD risk even if uric acid levels are still within normal limits [27,28]. The levels of UA predicting CVD varies from 5.1 to 5.7 mg/dL depending on the endpoint and sex [29,30].

There is still no sufficient explanation of the role of hyperuricemia as a risk factor for CAD in T1D patients. Some studies have shown that higher serum UA levels independently predict cardiovascular events in T1D [31]. In contrast, other researchers did not show a significant association between UA and coronary vascular function in patients with diabetes [32].

To our knowledge, studies evaluating the association between serum UA levels and coronary artery disease in pancreas transplant candidates have not yet been conducted. Pancreas recipients constitute a group of patients with a very high cardiovascular risk, so every effort should be made to reduce it. One of the most important steps in reducing cardiovascular risk is the identification of all potential risk factors. Elevated uric acid seems to be one of the potential risk factors for CAD in this group.

Therefore, the objective of this study was to evaluate the association between serum uric acid levels and the prevalence of coronary artery disease and other classical cardiovascular risk factors in patients eligible for pancreas transplantation.

## 2. Materials and Methods

### 2.1. Study Population

This cross-sectional study was conducted at the 3rd Department of Internal Medicine and Cardiology, Medical University of Warsaw (Poland). All patients with T1D eligible for pancreas transplantation (SPKT or PTA) who were referred to our center for cardiac evaluation before transplantation were prospectively included in the study. Patients were recruited from August 2018 to April 2021. The study protocol was approved by the local Bioethics Committee of the Medical University of Warsaw (Poland) (no. KB/115/2018). All participants signed an informed consent form to participate in the study.

### 2.2. Measurements and Definitions of Variables

The following demographic and medical data were collected: age, sex, the duration of T1D, renal replacement therapy, the main cardiovascular risk factors (hypertension, dyslipidemia, smoking habit), and type of planned transplantation procedure. Dyslipidemia was defined when total cholesterol (TC) was ≥5.2 mmol/L and/or low-density lipoprotein-cholesterol (LDL-C) was ≥3.0 mmol/L and/or triglycerides (TG) were ≥1.7 mmol/L or a patient was on lipid-lowering therapy. Smoking habit was defined as active smoking. Hypertension was defined when either a systolic blood pressure (SBP) or diastolic blood pressure (DBP) was above 140/90 mmHg and/or the antihypertensive medication was used before enrollment into the study. All patients were rated for using antihypertensive medication, statins, and pharmacological hypouricemic therapy during the last three months before the study entry.

Systolic blood pressure (SBP) and diastolic blood pressure (DBP) were measured in a seated position after 10 min of rest using an automatic oscillometric blood pressure monitor. Measurements were taken on 3 consecutive days and each measurement was repeated three times with at least a 5 min interval. The mean value of SBP and DBP was calculated as an average of 9 measurements (3 measurements over 3 days) and was considered as the patient’s blood pressure. Pulse pressure (PP) was defined as SBP–DBP.

Body mass index (BMI) was calculated as weight in kilograms divided by height in meters squared. Height (m) and weight (kg) were measured on the day of blood sampling. The weight of hemodialysis (HD) subjects was measured on the day of dialysis. Obesity was defined as a BMI ≥ 30 kg/m^2^.

Blood samples were collected following a 12 h overnight fast. A commercially available analyzer (Beckman Coulter, Inc. Brea, CA, USA) was used to measure total cholesterol (TC), high-density lipoprotein-cholesterol (HDL-C), triglycerides (TG), glycated hemoglobin (HbA1c), creatinine, and uric acid (UA) in plasma/serum. The concentration of low-density lipoprotein-cholesterol (LDL-C) was calculated using the Friedewald formula [33]. The serum urid acid/creatinine ratio (UA/Cr) was calculated by dividing the serum values of uric acid by creatinine. Glomerular filtration rate (eGFR) was estimated only in non-dialyzed participants using the Modification of Diet in Renal Disease Study (MDRD, 4-variable version) [34].

For comparative analyses, our study population was divided into two subgroups on the basis of UA levels (elevated/normal UA level). The results of the URRAH (Uric Acid Right for Heart Health) study were used to define the cut-off points for uric acid levels. Based on the URRAH study, the cut-off value for better discrimination of cardiovascular mortality was 5.1 mg/dL (95% CI, 4.34–5.70) in women, and 5.6 mg/dL (95% CI, 5.30–5.78) in men [35]. Therefore, in our study, an elevated UA level was defined as ≥5.1 mg/dL for females and ≥5.6 mg/dL for males, normal UA level was defined when the UA concentration was below the accepted cut-off points.

All participants underwent coronary angiography through a Philips Allura Xper DF20 X-ray system using standard diagnostic catheters. Radial access was routinely used and femoral access was used only in case of significant technical difficulties with radial access. The obstructive coronary artery disease was diagnosed when there was at least one stenosis ≥ 50% in at least one major epicardial artery.

### 2.3. Statistical Analysis

Categorical variables were presented descriptively by frequency and percentage distribution. The normality of the distribution of the data was tested using the Shapiro–Wilk test. For parameters not having normal distributions, the data were expressed as the median with interquartile range (IQR) and statistical analyses were based on non-parametric tests. The Mann–Whitney U test was used to compare numerical variables between the two groups, and the Fisher’s exact test or chi-squared test was used to examine the relationship between categorical variables. The statistical significance obtained from this test proves the existence of a relationship between two nominal variables.

The correlations between uric acid (UA) and numerical variables were assessed using the Spearman or Pearson correlation coefficient (r). Due to the presence of outliers, the Spearman correlation coefficient was calculated instead of the Pearson correlation coefficient. The following classification of the correlation strength was used: 0.0 ≤ |r| ≤ 0.2, no correlation; 0.2 ≤ |r| ≤ 0.4, low correlation; 0.4 ≤ |r| ≤ 0.7, moderate correlation; 0.7 ≤ |r| ≤ 0.9, high correlation; 0.9 ≤ |r| ≤ 1.0, very high correlation.

A multivariate logistic regression model was performed to test the combined relationship between the presence/absence of CAD and selected clinical and laboratory parameters. All variables that proved significant in univariate analysis (*p* < 0.05) were included in the multivariate model (TG, UA, SBP, PP, HD). Hypertension and statin intake were also included in the model as confounders potentially affecting CAD risk, despite the lack of a statistically significant effect on CAD (*p* < 0.1 was used as an inclusion value). eGFR was calculated only for non-HD patients and was not included in the multivariate analysis. Based on the Akaike information criterion (AIC), the best-fit model was selected. On the basis of the coefficients’ β, the value of their exponents, exp(β), was calculated, which is the unit odds ratio (OR). OR means the value of how many times the odds of an event described by the dependent variable will increase (or decrease) if the value of the given predictor increases by one.

Statistical analyses were performed using the R software for statistical computing (version 4.0.3, R Core Team 2020, Vienna, Austria). A *p*-value of <0.05 was considered to be significant for all statistical analyses.

## 3. Results

### 3.1. Participant Characteristics

The study population included 63 patients (30 males and 33 females; a median age of 41 (35–46.5) years) eligible for pancreas transplantation (SPKT or PTA). According to the transplant team’s decision, 71.42% of patients (*n* = 45) were qualified for SPKT and 28.57% of patients (*n* = 18) were qualified for PTA. The indications for PTA were as follows: brittle diabetes (*n* = 15; 83.34% out of 18), rapid progression of microangiopathic complications (*n* = 2; 11.11%), and severe insulin therapy-related emotional problems in one patient. T1D patients with end-stage diabetic kidney disease qualified for SPKT. The majority of SPKT candidates were on hemodialysis (*n* = 39; 86.67% out of SPKT group) and others (*n* = 6; 13.33%) qualified for SPKT as a pre-emptive transplantation. The baseline characteristics of the participants are illustrated in Table 1.

The median duration of T1D was 26 (21.5–31) years. The duration of diabetes exceeded 20 years in more than three quarters (*n* = 49; 77.78%) of the study participants. End-stage renal disease was found in 71.42% of participants (*n* = 45). The majority of patients were on hemodialysis (HD) (*n* = 39; 61.9% of the entire cohort) with a median duration time of HD 24 (15–34.5) months. CAD was present in 23 (36.5%) participants. The prevalence of traditional cardiovascular disease (CVD) risk factors (hypertension, dyslipidemia, smoking habit, obesity) was very high. At least one additional CVD risk factor (except for T1D) was found in 55 (87.3%) participants, and patients with two risk factors for coronary heart disease comprised more than one-third of the study cohort (*n* = 23; 36.5%). Finally, only eight (12.7%) patients had no classical CVD risk factors except for T1D. The most common CVD risk factors in our cohort were hypertension (*n* = 49; 77.8%) and dyslipidemia (*n* = 44; 69.8%). The majority of patients (*n* = 48; 76.2 %) reported the use of antihypertensive medication, and almost half of the participants (*n* = 31; 49.2%) reported the use of statins. Two participants (3.2%) reported the use of hypouricemic drugs three months before the study entry.

### 3.2. Uric Acid Concentration and Its Association with CAD

**Figure 1:**
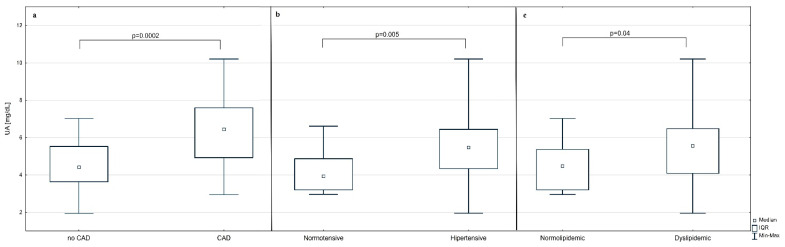
The concentrations of the uric acid in participants with and without analyzed comorbidities. (**a**) Patients with coronary artery disease (CAD) vs. patients without CAD (no CAD). (**b**) Hypertensive patients vs. normotensive patients. (**c**) Dyslipidemic patients vs. normolipidemic patients.

The median concentration of uric acid in the whole study group was 4.92 (3.94–6.34) mg/dL. The associations between the UA concentration and CAD, hypertension, and dyslipidemia were proved to be significant using the Mann–Whitney U test. The median concentration of UA in patients with CAD was higher by 2.02 mg/dL than in participants without CAD (*p* = 0.0002) (Figure 1). Similarly, the median UA concentrations in patients with hypertension or dyslipidemia were significantly higher than in participants without these comorbidities (5.47 (4.33–6.43) vs. 3.93 (3.24–4.79) mg/dL, *p* = 0.005 and 5.55 (4.12–6.45) vs. 4.47 (3.29–5.29) mg/dL, *p* = 0.04, respectively).

We did not find any significant relationships between UA concentration and other clinical variables (sex, smoking habit, HD) (Supplementary Table S1). Spearman’s and Pearson’s correlation analyses were used for testing the correlations between UA concentration and selected laboratory and clinical parameters. We proved the statistically significant correlations between the concentration of UA and SBP, PP, eGFR, and TG. The correlation between the UA concentration and eGFR was negative (r = −0.534, *p* = 0.007), i.e., when the eGFR value decreases, the UA concentration increase. This correlation was of moderate strength. The correlations between UA concentration and SBP, PP, and TG were positive and had low strength. We did not prove any significant correlations between the concentration of UA and the other analyzed parameters (age, duration of T1D, duration of hemodialysis, BMI, TC, LDL-C, HDL-C, HbA1c, DBP) (Table 2).

### 3.3. Relationship between Elevated/Normal UA Levels and CAD

Elevated UA levels were found in about two-thirds of patients with CAD (15 out of 23; 65.2%) and only in approximately one-third of participants without CAD (11 out of 40; 27.5%). The prevalence of CAD was significantly higher in patients with elevated UA levels than in those with normal levels (57.7% vs. 21.6%, *p* = 0.008). Furthermore, the prevalence of hypertension or dyslipidemia was significantly higher in patients with elevated UA levels than in patients with normal UA levels (92.3% vs. 67.6%, *p* = 0.03 and 88.5% vs. 56.8%, *p* = 0.01; respectively) (Table 1). There were no significant differences in age, sex, BMI, the duration of T1D, and HbA1c between the two analyzed subgroups.

Moreover, we did not find any significant difference in UA levels between hemodialyzed and non-hemodialyzed patients, as well as no difference in UA levels related to the duration of HD. In non-hemodialyzed patients, a significant relationship between UA levels and eGFR was proven using the Mann–Whitney U test. The median values of eGFR (*n* = 24) were significantly lower in patients with elevated UA levels than in patients with low UA levels (19.75 (14.55–31.68) vs. 91.65 (86.03–114.57) mL/min/1.73 m^2^, *p* = 0.005). TG concentration was the only lipid parameter that differed significantly in the analyzed subgroups; the median TG concentration was 42.3% higher in subjects with elevated UA levels than in normal UA levels (1.85 (1.25–2.08) vs. 1.3 (1–1.8) mmol/L, *p* = 0.006). We did not find any significant differences in TC, LDL-C, and HDL-C between subgroups divided according to elevated/low UA levels.

### 3.4. Relationship between CAD and Selected Clinical and Laboratory Parameters

CAD was found in 23 (36.5%) patients. The characteristics of participants divided into subgroups according to the presence/absence of CAD are illustrated in Table 3. Patients with CAD had significantly higher SBP (145 (127–158) vs. 131 (124.5–138) mmHg, *p* = 0.02), PP (63 (59–74) vs. 53 (48–59.5) mmHg, *p* = 0.002), TG (1.8 (1.5–2.1) vs. 1.2 (0.95–1.7) mmol/L, *p* = 0.0004), and UA concentration (6.43 (4.92–7.58) vs. 4.41 (3.63–5.52) mg/dL, *p* = 0.0002) than patients without CAD. There were no significant differences in age, sex, and BMI between the two analyzed subgroups. Diabetes-specific factors (HbA1c and diabetes duration) and kidney disease-specific factors (HD, eGFR) also did not have any significant associations in relation to the prevalence of CAD.

In the univariate logistic regression analysis, the concentration of UA (OR = 2.14; 95% CI: 1.373–3.337, *p* = 0.0008), TG concentration (OR 3.986; 95% CI: 1.472–10.791, *p* = 0.006), values of SBP (OR 1.046; 95% CI: 1.010–1.084, *p* = 0.012) and PP (OR 1.091; 95% CI: 1.030–1.156, *p* = 0.003), and renal replacement therapy (OR 3.257; 95% CI: 1.012–10.485, *p* = 0.048) were significantly associated with CAD (Table 4).

The multivariate analysis demonstrated that the concentration of UA (OR 2.044; 95% CI: 1.261–3.311, *p* = 0.004) and the values of PP (OR 1.077; 95% CI: 1.009–1.15, *p* = 0.03) were independently associated with the prevalence of CAD in the presented cohort.

## 4. Discussion

The main finding of our study is that elevated UA levels are strongly associated with a prevalence of CAD in T1D patients eligible for pancreas transplantation. In addition, we demonstrated a significant association between elevated UA, hypertension, and dyslipidemia, which are common CVD risk factors and typical comorbidities in this group of patients. Finally, a concentration of UA and the values of PP were found to be the independent factors associated with the prevalence of CAD in the presented cohort.

Our study evaluated a T1D population with long-standing diabetes and multiple traditional CVD risk factors. The prevalence of CAD (36.5%) in our group was in line with the results of other researchers [6,7,36]. In our study, only 41.2% of participants demonstrated elevated UA levels. However, CAD was significantly more common in people with elevated uric acid than in those with normal levels. Coronary angiography revealed CAD in almost two-thirds of patients with elevated UA, while only in approximately one-fifth of participants with normal UA levels. Furthermore, we proved that the median UA concentration was 45.8% higher in patients with CAD than in patients without CAD. The most impressive result of this study is that the concentration of UA was found to be an independent predictor for the prevalence of CAD in our cohort. When the concentration of UA increased by 1 mg/dL, the odds of having CAD increased by 2.04 times. Regarding the association between UA and CAD, the results of our study are consistent with the results of other researchers. Kivity S et al. demonstrated that the occurrence of cardiovascular events positively correlates with UA levels, and concluded that serum UA can be used as a marker of CVD risk in a healthy population [37]. According to Yu J et al., UA is not only a marker of CAD but also a marker of its severity [38]. They found that UA levels positively correlated with the Syntax score and with the number of diseased vessels in 347 patients with obstructive coronary artery disease. Furthermore, Li L et al. demonstrated that patients with an early onset of hyperuricemia (<45 years) had an increased risk of cardiovascular disease and all-cause mortality in comparison to the control group [39]. In our study, the median age of the participants was 41 (35–46.5) years, so our results are consistent with that reported by Li L et al.

Despite the large number of studies evaluating the association of hyperuricemia with CAD in the general population, the association between UA and CAD in patients qualified for pancreas transplantation has not yet been investigated. However, several studies have evaluated the relationship between UA and CAD in the overall population of T1D patients. Rodrigues et al. demonstrated that elevated UA levels predicted the progression of coronary artery calcification (CAC) in T1D patients without renal disease [40]. Similarly, Bjornstad et al. demonstrated that serum UA independently predicted the development of CAC (OR 1.5, 95% CI: 1.1–1.9) in adult T1D patients in 6 years of follow-up. Moreover, this association was evident despite that uric acid levels remained within the normal range in most participants [41]. In this regard, the results we presented are consistent with the study discussed above. In our study, a significant association between UA and CAD was proven, even though UA levels remained within normal limits in most subjects.

In addition to studies determining the relationship between UA and subclinical markers of atherosclerosis, there are also some studies evaluating the association between UA and cardiovascular events. Pilemann-Lyberg et al. demonstrated that increased uric acid levels were a significant and independent risk factor for cardiovascular events in T1D patients during 5.1 (4.7–5.6) years of follow-up [31]. However, in a cross-sectional study performed by Rathmann et al., the elevated serum uric acid levels (UA concentrations > 7.0 mg/dL in men and >6.6 mg/dL in women) correlated with the presence of coronary heart disease in diabetic women rather than in men [42]. Similarly, Jenkins et al. demonstrated the association between elevated UA levels and the major adverse cardiovascular events in women with T1D [32]. However, the analysis of the entire cohort (both sexes) of participants with T1D did not show any significant associations between UA and subsequent CVD. Thus, the authors concluded that there is no justification for the routine measurement of UA levels to assess CVD risk in patients with T1D. The results of our study are opposite to the above results. One explanation for this discrepancy is that the group we studied was different from the cohort described by Jenkins et al. Our study evaluated a population with a wider range of renal functions. In our study, the majority of participants presented the end-stage renal disease (*n* = 45, 71.42%), of whom 39 (61.9% of the entire cohort) were on hemodialysis. Moreover, in our group, the duration of T1D was longer and the frequency of traditional CVD risk factors (hypertension, dyslipidemia, smoking habit) was higher. In addition, the cut-off points for UA concentrations were significantly different, resulting in a significantly higher proportion of subjects with elevated UA concentrations.

Generally, the conclusions of studies evaluating the association of hyperuricemia with CAD in the T1D population are not fully consistent. Conflicting results may be due to many reasons, such as a difference in enrolled subjects (age, gender, comorbidities), cut-off points for hyperuricemia, different endpoints, and study design. Moreover, the relationship between UA and CAD can be exaggerated by other established risk factors or other undetected confounding factors [43]. Genetic evidence based on Mendelian randomization studies did not support a relevant causal effect of genetically predicted UA levels on CAD in patients with diabetes [44]. However, Efstathiadou et al. concluded that the weak association of genetically determined UA with CAD may result from pleiotropic effects [45]. It is worth noting that the UA metabolism changes during kidney disease in T1D. Decreased glomerular filtration, reduced tubular secretion, and enhanced tubular reabsorption leads to decreased excretion of uric acid in kidneys. Both glomerular and tubular injury are the recognized complication of diabetic ketoacidosis [46]. Under ischemic conditions, uric acid may act as a pro-oxidant factor promoting endothelial dysfunction and atherosclerosis [12].

In our study, the prevalence of traditional CVD risk factors was very high; hypertension or dyslipidemia was found in the majority of participants and only 12.7% of patients had no classical CVD risk factors except for T1D. We demonstrated that the median UA concentrations in patients with hypertension or dyslipidemia were significantly higher than in the other participants. Furthermore, the prevalence of hypertension or dyslipidemia was significantly higher in patients with elevated UA levels than in patients with normal UA levels. Elevated UA levels are intimately associated with hypertension [22]. There are several possible mechanisms for this association including the upregulation of the renin–angiotensin–aldosterone system, kidney afferent arteriolopathy, UA-induced oxidative stress and systemic inflammation [47].

To further explain the relationship between the concentration of UA and CAD, we examined the correlations between UA and the other CVD risk factors: the values of blood pressure and lipid profile parameters. Of the lipid profile parameters we analyzed, only TG correlated significantly with UA concentration, and the median TG concentration was significantly higher in subjects with elevated UA levels than in normal UA levels. Our findings are in line with the results of Jenkins et al. who showed the strong association between UA and TG in the entire cohort of early middle-aged patients with T1D [32]. In our study, we found statistically significant positive correlations between the concentration of UA and values of SBP and PP. In this regard, our study is consistent with the results of Zhang et al., who demonstrated a strong and positive association between UA and SBP in the general population [48]. In contrast, Jenkins et al. found that higher UA levels were associated with higher SBP only in T1D men, while the prevalence of hypertension was higher only in T1D women [32]. It should be noted, however, that a direct comparison of their results to ours is not easy. The upper limits for normal UA concentrations in our study were lower than in the mentioned studies, and the small group size did not allow us to perform reliable sex-specific analyses. In our study, the values of PP were significantly associated with the prevalence of CAD as well. PP is inversely proportional to arterial compliance and has a strong correlation with pulse wave velocity, which is considered to be an index of arterial stiffness [49]. UA may lead to arterial stiffening as a consequence of oxidative stress and chronic vascular inflammation. UA decreases endothelial nitric oxide (NO) synthase activity and NO production [50], stimulates vascular smooth muscle cell proliferation [51], and increases the expression of cyclooxygenase 2 and the production of angiotensin II [52]. Arterial stiffness leads to systolic hypertension [53] and predicts cardiovascular events in the general population [54,55,56]. Furthermore, Llauradó et al. demonstrated that arterial stiffness was highly correlated with the Steno Type 1 Risk Engine scores [57], which is a specific CVD risk-estimation tool for the T1D population [58]. The mentioned authors concluded that the measurement of arterial stiffness could be used as a tool to assess cardiovascular risk in T1D. The results presented by Llauradó confirm the practical significance of our observations.

The association between UA and CAD in CKD patients has been a topic of increased interest in recent years. Serum UA level may serve as a marker of decreased renal function with reduced eGFR and as predictor of the severity of coronary artery stenosis among non-dialyzed patients [59]. However, the results of studies investigating the association between UA and cardiovascular disease in hemodialyzed patients are not consistent [60,61]. In our study, we found a significant inverse correlation between eGFR and UA concentration in non-hemodialyzed patients. Otherwise, in the univariate analysis, we did not find a significant association between eGFR and CAD in non-hemodialyzed patients. There are several factors (low age, long-standing T1D and multiple traditional CVD risk factors) that could probably confound the relationship between eGFR and CAD in the non-hemodialyzed subgroup. In our study, the majority of patients were on replacement therapy. We did not find any significant difference in UA levels between hemodialyzed and non-hemodialyzed patients, as well as no difference in UA levels related to the duration of HD. Since our population included the participants with chronic kidney disease, we performed an additional analysis on serum uric acid to creatinine ratio (UA/Cr). According to Al-Daghri NM et al., the UA/Cr ratio was found to be strongly associated with metabolic syndrome and its components in non-dialyzed patients with type 2 diabetes mellitus [62]. In our study, we did not find any association between the UA/Cr ratio and CAD; this could be due to a very high percentage of hemodialyzed participants. In the univariate analysis, renal replacement therapy was found to be significantly associated with the prevalence of CAD. However, in the multivariate analysis, only the concentration of UA and the values of PP were independently associated with the prevalence of CAD. Finally, the study we presented found a significant association between UA and the prevalence of CAD in the entire group of pancreas transplant candidates (SPKT or PTA).

Our study has some limitations. First, due to the nature of the presented study (cross-sectional), no causal relationships can be established. Elevated uric acid may account for the high prevalence of coronary heart disease, as well as being only a significant marker of it. The second limitation is the lack of a non-diabetic control group. The third limitation is a small sample size. The main reason for this is that pancreas transplantation is a procedure performed infrequently in Poland (about 30 transplantations per year) with a significant decline in this procedure in 2020 due to the SARS-CoV-2 epidemic [63]. Therefore, the number of patients enrolled for this method of treatment is adequately small. A significant limitation of the study is that we focused on diagnosing an obstructive CAD. Due to a high clinical likelihood of CAD, our patients were proceeded directly to invasive coronary angiography. Invasive diagnostic tools to investigate microcirculatory or vasomotor coronary disorders are not commonly used and were not implicated in our study. Another limitation is the use of statins and a lack of details on antihypertensive drugs, specifically RAAS inhibitors, which may have confounded the presented relationships. However, the study aimed to test the real population of pancreas transplant candidates, so we included all patients without any selection bias.

Despite its limitation, our study has several strengths, and its conclusions can be applied in everyday practice. To our knowledge, this is the first study exploring the relationship between UA and CAD in T1D pancreas transplant candidates. We enrolled all consecutive patients referred to our center for cardiological evaluation prior to surgery (PTA or SPKT), so our study results reflect a real population of patients eligible for pancreas transplantation. In the presented study, the majority of participants were patients with long-standing diabetes with many CVD risks factors and more than half of participants were on hemodialysis. It is worth noting that CAD was found in 36.5% of patients despite being angina-asymptomatic. Taken together, our cohort constitutes a high-risk group in respect to perioperative cardiac complications. Therefore, the identification of additional, non-classical CVD risk factors in this population is very important. We have shown that an elevated UA level was an independent factor significantly associated with a higher prevalence of CAD and strongly associated with CVD risk factors (hypertension, dyslipidemia), all considered to be an important cause of perioperative cardiovascular complications. From a practical point of view, we suggest that UA should be evaluated to stratify CVD risk in T1D patients eligible for pancreas transplantation, regardless of whether it is a risk factor or just a marker of CAD in this group of patients.

## 5. Conclusions

Elevated UA levels are strongly associated with a high prevalence of CAD in T1D patients eligible for pancreas transplantation. In addition, there is a significant relationship between elevated UA and hypertension and dyslipidemia. To stratify CVD risk, the measurement of the UA concentration should be considered in all T1D patients qualified for pancreas transplantation. Further studies are needed to confirm the cause-and-effect relationship between an elevated UA level and CAD in pancreas transplant candidates with T1D.

## Figures and Tables

**Table 1:** Characteristics of the study group stratified by the uric acid levels (elevated/normal).

Variable	Total (N = 63)	Elevated UA Level (N = 26)	Normal UA Level (N = 37)	*p*-Value
Age (years)	41 (35–46.5)	40.5 (33–48.75)	41 (36–46)	0.8
Sex (Female)	33 (52.4%)	13 (50%)	20 (54%)	0.95
Duration of T1D (years)	26 (21.5–31)	26.5 (24.25–33)	25 (21–29)	0.4
Hemodialyzed patients	39 (61.9%)	18 (69.2%)	21 (56.8%)	0.5
Duration of HD (months)	24 (15–34.5); N = 39	24 (17.25–34); N = 18	26 (14–33); N = 21	0.98
BMI (kg/m^2^)	22.77 (20.82–24.49)	21.3 (20.53–26.32)	22.94 (21.36–24.24)	0.6
Hypertension	49 (77.8%)	24 (92.3%)	25 (67.6%)	**0.03**
Dyslipidemia	44 (69.8%)	23 (88.5%)	21 (56.8%)	**0.01**
Statin intake	31 (49.2%)	17 (65.4%)	14 (37.8%)	0.06
Smoking habit	19 (30.2%)	8 (30.8%)	11 (29.7%)	1.0
CAD	23 (36.5%)	15 (57.7%)	8 (21.6%)	**0.008**
SBP (mmHg)	132 (125–148.5)	137.5 (129–150.25)	131 (124–147)	0.2
DBP (mmHg)	77 (72–83.5)	77 (71.25–83.75)	77 (73–83)	0.9
PP (mmHg)	58 (50–66)	60 (54–67.25)	53 (47–64)	0.06
HbA1c (%)	7.61 (7.11–8.84)	8.31 (7.25–9.52)	7.47 (6.95–8.22)	0.1
eGFR (mL/min/1.73 m^2^)	88.1 (26.63–109.1); N = 24	19.75 (14.55–31.68); N = 8	91.65 (86.03–114.57); N = 16	**0.005**
TC (mmol/L)	4.7 (3.65–5.6)	4.95 (3.73–5.65)	4.3 (3.6–5.1)	0.3
LDL-C (mmol/L)	2.5 (1.95–2.95)	2.65 (1.9–2.98)	2.4 (2–2.8)	0.6
HDL-C (mmol/L)	1.4 (1.2–1.6)	1.35 (1.2–1.6)	1.4 (1.2–1.6)	0.9
TG (mmol/L)	1.5 (1.1–1.9)	1.85 (1.25–2.08)	1.3 (1–1.8)	**0.006**
UA (mg/dL)	4.92 (3.94–6.34)	N/A	N/A	N/A

Elevated UA level was defined as ≥5.1 mg/dL for females and ≥5.6 mg/dL for males, low UA level was defined when the UA concentration was below the accepted cut-off points. Categorical variables are presented as number and percentage (%), and continuous variables are presented as the median with interquartile range (IQR). T1D, type 1 diabetes; HD, hemodialysis; BMI, body mass index; CAD, coronary artery disease; SBP, systolic blood pressure; DBP, diastolic blood pressure; PP, pulse pressure; HbA1c, glycated hemoglobin; eGFR, estimated glomerular filtration rate calculated in non-hemodialyzed patients using the MDRD formula (see Materials and Methods); TC, total cholesterol; LDL-C, low-density lipoprotein cholesterol; HDL-C, high-density lipoprotein cholesterol; TG, triglycerides; UA, uric acid; N/A, non-applicable. Significant differences are marked in bold.

**Table 2:** Correlations between uric acid concentration and selected clinical and laboratory parameters (N = 63).

Variable	Correlation Coefficient (r)	*p*-Value
Age (years)	0.129 *	0.314
Duration of T1D (years)	0.079 *	0.540
Duration of HD (months); N = 39	−0.037 **	0.825
BMI (kg/m^2^)	0.022 **	0.862
SBP (mmHg)	0.271 *	**0.032**
DBP (mmHg)	0.081 *	0.528
PP (mmHg)	0.327 **	**0.009**
eGFR (mL/min/1.73 m^2^); N = 24	−0.534 *	**0.007**
TC (mmol/L)	0.147 *	0.249
LDL-C (mmol/L)	0.067 *	0.601
HDL-C (mmol/L)	−0.005 **	0.970
TG (mmol/L)	0.354 **	**0.004**
HbA1c (%)	0.166 **	0.195

(r) *, Pearson’s correlation coefficient; (r) **, Spearman’s correlation coefficient; T1D, type 1 diabetes; HD, hemodialysis; BMI, body mass index; SBP, systolic blood pressure; DBP, diastolic blood pressure; PP, pulse pressure; eGFR, estimated glomerular filtration rate calculated in non-hemodialyzed patients using the MDRD formula (see Materials and Methods); TC, total cholesterol; LDL-C, low-density lipoprotein cholesterol; HDL-C, high-density lipoprotein cholesterol; TG, triglycerides; HbA1c, glycated hemoglobin. Significant differences are marked in bold.

**Table 3:** Characteristics of the study group stratified by presence/absence of coronary artery disease.

Variable	Total (N = 63)	CAD (N = 23)	No CAD (N = 40)	*p*-Value
Age (years)	41 (35–46.5)	43 (37–51)	40 (34.5–46)	0.2
Sex (Female)	33 (52.4%)	13 (56.5%)	20 (50%)	0.8
Duration of T1D (years)	26 (21.5–31)	27 (24–34)	25.5 (20.5–29)	0.3
Hemodialyzed patients	39 (61.9%)	18 (78.3%)	21 (52.5%)	0.06
Duration of HD (months)	24 (15–34.5); N = 39	27.5 (18–36); N = 18	22 (10–27); N = 21	0.06
BMI (kg/m^2^)	22.77 (20.82–24.49)	21.4 (20.2–25.1)	22.8 (20.9–24.4)	0.8
Hypertension	49 (77.8%)	21 (91.3%)	28 (70%)	0.06
Dyslipidemia	44 (69.8%)	19 (82.6%)	25 (62.5%)	0.2
Statin intake	31 (49.2%)	15 (65.2%)	16 (40%)	0.07
Smoking habit	19 (30.2%)	9 (39.1%)	10 (25%)	0.3
CAD	23 (36.5%)	N/A	N/A	N/A
SBP (mmHg)	132 (125–148.5)	145 (127–158)	131 (124.5–138)	**0.02**
DBP (mmHg)	77 (72–83.5)	78 (71–88)	76.5 (72.5–83.5)	0.5
PP (mmHg)	58 (50–66)	63 (59–74)	53 (48–59.5)	**0.002**
HbA1c (%)	7.61 (7.11–8.84)	7.82 (7.21–9.3)	7.46 (6.895–8.64)	0.1
eGFR (mL/min/1.73 m^2^)	88.1 (26.63–109.1); N = 24	28.6 (20.7–40.9); N = 5	91 (33.8–118.4); N = 19	0.08
TC (mmol/L)	4.7 (3.65–5.6)	5 (3.6–5.7)	4.35 (3.65–5.45)	0.4
LDL-C (mmol/L)	2.5 (1.95–2.95)	2.7 (1.8–3.2)	2.45 (1.95–2.8)	0.4
HDL-C (mmol/L)	1.4 (1.2–1.6)	1.3 (1.2–1.5)	1.4 (1.2–1.7)	0.4
TG (mmol/L)	1.5 (1.1–1.9)	1.8 (1.5–2.1)	1.2 (0.95–1.7)	**0.0004**
UA (mg/dL)	4.92 (3.94–6.34)	6.43 (4.92–7.58)	4.41 (3.63–5.52)	**0.0002**
UA/Cr	1.09 (0.64–3.50)	1.022 (0.76–1.94)	1.26 (0.62–4.73)	0.4

Categorical variables are presented as number and percentage (%), and continuous variables are presented as the median with interquartile range (IQR). T1D, type 1 diabetes; HD, hemodialysis; BMI, body mass index; CAD, coronary artery disease; SBP, systolic blood pressure; DBP, diastolic blood pressure; PP, pulse pressure; HbA1c, glycated hemoglobin; eGFR, estimated glomerular filtration rate calculated in non-hemodialyzed patients using the MDRD formula (see Materials and Methods); TC, total cholesterol; LDL-C, low-density lipoprotein cholesterol; HDL-C, high-density lipoprotein cholesterol; TG, triglycerides; UA, uric acid; UA/Cr, serum urid acid to creatinine ratio; N/A, non-applicable. Significant differences are marked in bold.

**Table 4:** Univariate and multivariate logistic regression analyses of factors associated with CAD (N = 63).

Univariate Logistic Regression Analysis				
Variable	β	OR	95% CI	*p*-Value
Sex (Male)	−0.262	0.769	0.274–2.158	0.6
Age	0.047	1.049	0.979–1.123	0.2
Hypertension	1.504	4.500	0.908–22.296	0.066
BMI (kg/m^2^)	0.012	1.012	0.878–1.166	0.9
Statin intake	1.034	2.812	0.969–8.167	0.057
Smoking	0.657	1.929	0.641–5.803	0.2
eGFR (mL/min/1.73 m^2^) *	−0.028	0.973	0.945–1.001	0.063
LDL-C (mmol/L)	0.312	1.367	0.712–2.623	0.3
TG (mmol/L)	1.383	3.986	1.472–10.791	**0.007**
UA (mg/dL)	0.761	2.140	1.373–3.337	**0.0008**
UA/Cr	−0.290	0.749	0.546–1.026	0.072
SBP (mmHg)	0.045	1.046	1.010–1.084	**0.01**
DBP (mmHg)	0.032	1.032	0.973–1.096	0.3
PP (mmHg)	0.087	1.091	1.030–1.156	**0.003**
HD	1.181	3.257	1.012–10.485	**0.048**
Multivariate logistic regression analysis				
UA (mg/dL)	0.715	2.044	1.261–3.311	**0.004**
PP (mmHg)	0.074	1.077	1.009–1.15	**0.03**

The multivariate model of CAD included all the variables that were significant in the univariate analysis (UA, TG, SBP, PP, HD) and potential confounding factors (hypertension and statin intake). Based on the Akaike information criterion (AIC) the best-fit model was selected. On the basis of the coefficients’ β, the value of their exponents, exp(β), was calculated, which is the unit odds ratio. *, N = 24; CAD, coronary artery disease; OR, odds ratio; CI, confidence interval; BMI, body mass index; eGFR, estimated glomerular filtration rate calculated in non-hemodialyzed patients using the MDRD formula (see Materials and Methods); LDL-C, low-density lipoprotein cholesterol; TG, triglycerides; UA, uric acid; UA/Cr, serum urid acid to creatinine ratio; SBP, systolic blood pressure; DBP, diastolic blood pressure; PP, pulse pressure; HD, hemodialysis. Significant differences are marked in bold.

## Data Availability

The data presented in this study are available on request from the corresponding author.

## Supplementary Materials

The following supporting information can be downloaded at: https://www.mdpi.com/article/10.3390/jcm11092421/s1, Table S1: The comparison of uric acid concentrations in the study group according to sex, smoking habit, and hemodialysis.
