# Supplementary material for: The Impact of an Elevated Uric Acid Level on the Prevalence of Coronary Artery Disease in Pancreas Transplant Candidates with Type 1 Diabetes: A Cross Sectional Study
Source: J Clin Med. 2022 Apr 26;11(9):2421. doi: 10.3390/jcm11092421 (PMC9102555; doi:10.3390/jcm11092421)
Supplement: Supplementary file 1 [file jcm-11-02421-s001.zip › jcm-1603598-supplementary.pdf]

Table S1. The comparison of uric acid concentrations in the study group according to sex, hemodialysis and smoking habit.

Variable	Parameter		p-value
UA [mg/dl]	Males (N=30)	Females (N=33)	0.17
	5.36 (4.46 - 6.34)	4.47 (3.63 - 6.17)	
	HD patients (N=39)	Non-HD patients (N=24)	0.13
	5.37 (4.28 - 6.42)	4.49 (3.58 - 5.67)	
	Smokers (N=19)	Non-smokers (N=44)	0.3
	5.22 (4.28 - 6.85)	4.9 (3.79 - 6.21)	

UA, uric acid; HD, hemodialysis. Data are presented as median with interquartile range (IQR).
